# Hepatic artery pseudoaneurysm–the Mayo Clinic experience and literature review

**DOI:** 10.3389/fmed.2024.1484966

**Published:** 2024-12-10

**Authors:** Tatjana Gavrancic, Muhammad Waqas Tahir, Marko Gorasevic, Igor Dumic, Libardo Rueda Prada, Melissa Cortes, Patricia Chipi, Zlatko Devcic, Charles Ritchie, Aleksandra Murawska Baptista

**Affiliations:** ^1^Department of Hospital Internal Medicine, Mayo Clinic, Jacksonville, FL, United States; ^2^Department of Research, Mayo Clinic, Jacksonville, FL, United States; ^3^Department of Hospital Internal Medicine, Mayo Clinic Health System, Eau Claire, WI, United States; ^4^Department of Interventional Radiology, Mayo Clinic, Jacksonville, FL, United States

**Keywords:** hepatic artery pseudoaneurysm, gastrointestinal bleeding, liver transplantation, infection, rupture

## Abstract

**Introduction:**

Hepatic artery pseudoaneurysm (HAP) is a rare and potentially life-threatening condition associated with high mortality. This study aims to review the etiology, clinical manifestations, management, and outcomes of patients diagnosed and treated for HAP at the Mayo Clinic.

**Methodology:**

This study was a retrospective chart review of medical records for patients diagnosed and treated for hepatic artery pseudoaneurysm (HAP) at the Mayo Clinic (Florida, Minnesota, and Arizona) between September 1, 1998, and June 30, 2022. A total of 27 patients with HAP were identified, and their demographics, presenting symptoms, location of HAP, etiology, associated liver pathology, type of intervention, and outcomes were analyzed.

**Results:**

The majority of patients with hepatic artery pseudoaneurysm (HAP) were male (63%), with a median age of 57 years (range: 25–87 years). HAP was predominantly intrahepatic (85.2%) and most commonly located on the right hepatic artery (RHA) (70.4%). In 89.9% of cases, the condition was attributable to hepatobiliary procedures or trauma, while only 10.1% occurred spontaneously. Presenting symptoms at the time of HAP diagnosis varied, including gastrointestinal (GI) bleeding (29.6%), abdominal pain (14.81%), non-GI bleeding (11.1%), traumatic bodily injury (11.1%), and other symptoms (14.81%). Asymptomatic or incidental findings of HAP were observed in 18% of cases. Malignancy was identified in 52% of patients, and 26% were liver transplant recipients. Statistical analysis revealed that factors such as prior knowledge of HAP (*p* = 0.381), HAP rupture (*p* = 0.382), anticoagulation therapy (*p* = 0.856), hemorrhagic shock (*p* = 0.25), liver cirrhosis (*p* = 0.143), gastrointestinal bleeding (*p* = 0.879), hepatobiliary abscess (*p* = 0.079), liver transplantation (*p* = 0.738), spontaneous HAP (*p* = 0.381), and malignancy (*p* = 0.163) were not significantly associated with increased mortality. In contrast, the need for transfusions (*p* = 0.021), tumor invasion (*p* = 0.023), portal vein thrombosis (PVT) (*p* = 0.02), and liver necrosis (*p* = 0.02) were significantly associated with higher mortality. The overall infection rate was 3%, while the mortality rate was 18.5%.

**Discussion:**

Hepatic artery pseudoaneurysm (HAP) is a rare but serious condition often associated with hepatobiliary procedures, trauma, or liver transplants, though it can also occur spontaneously. While HAP is commonly detected incidentally, its diagnosis is frequently linked to complications such as rupture and gastrointestinal bleeding. However, our study suggests that these complications do not necessarily increase mortality. Key factors associated with higher mortality include the need for blood transfusions, tumor invasion, portal vein thrombosis, and liver necrosis at the time of diagnosis. The overall infection rate was low, but the mortality rate was 18.5%, highlighting the importance of early detection and management.

## Introduction

Hepatic artery pseudoaneurysm (HAP) is a rare entity and usually described after it ruptures and causes life-threatening gastrointestinal bleeding. Its real incidence is unknown with some reports stating incidence as low as 0.001% (1). HAP occurs when there is loss of integrity of the arterial wall and formation of a false lumen between the tunica media and adventitia of the hepatic artery. Most commonly, this occurs due to atherosclerosis, mechanical injury of the arterial wall due to abdominal trauma or iatrogenic trauma during hepatobiliary procedures, acquired inflammatory process such as vasculitis, or by visceral inflammation or infiltration to the arterial wall from malignancy and, infection, such as pancreatitis, cholangitis, or cholecystitis. Only a small number of cases are classified as spontaneous due to unidentified specific factors. HAP can be anatomically divided into intrahepatic and extrahepatic. HAP following liver transplantation are most commonly extrahepatic (2), while the less common intrahepatic HAP occurs following liver biopsy (3). Clinical presentation varies from asymptomatic and incidentally found to severe gastrointestinal hemorrhage leading to hemorrhagic shock. HAP rupture can cause bleeding into the retroperitoneal space, stomach, or duodenum, venous, portal, or biliary system (presenting as Quicke’s triad with jaundice, biliary colic, and gastrointestinal bleeding) (3). HAP requires elective or emergent hepatic artery repair or ligation irrespective of its symptoms, size or location due to its substantial risk of rupture.

This study’s aim was to describe HAP presentation, etiology, associated liver pathologies, treatment options and mortality at Mayo Clinic.

## Methodology

We conducted a retrospective chart review of patients seen at The Mayo Clinic (Jacksonville, Florida; Rochester, Minnesota; and Phoenix, Arizona) between September 1^st^, 1998, and June 30th, 2022. This study was deemed minimal risk and exempt from IRB given its retrospective nature. All patients’ data were deidentified and collected in the excel spreadsheet. The Interventional Radiology department conducted a chart query to identify all patients who had a hepatic artery pseudoaneurysm (HAP) found on CT angiogram radiology reports that mentioned HAP with and without intervention. The final analysis included 27 cases of HAP. Each patient was counted only once, even if they had more than one admission with HAP. For these patients, the index event was the admission for the first HAP treatment.

Patients’ data were collected in excel spreadsheet and included the following: presenting symptoms, laboratory values (including white blood cell count, platelet count, liver function test, and INR) on the day of the index admission, hemodynamic stability (presence or absence of hemorrhagic shock), transfusion requirements as well as if patient was on anticoagulation when HAP was found. We further extracted data regarding the presence of liver necrosis, liver abscess, cholangitis, liver cirrhosis, portal venous thrombosis and, in patients with malignancy if there was tumor invasion causing HAP. Etiology was categorized as surgical (liver transplant or non-liver transplant), non-surgical procedure related, infection/tumor related, trauma or spontaneous. Spontaneous HAP was defined when there was no recent hepatobiliary procedure (liver biopsy, biliary drain placement, Whipple surgery), intrabdominal infection (e.g., cholangitis, pancreatitis), tumor (primary or metastatic), or trauma. HAP etiology was considered a surgical complication if it occurred within 90 days of any type of liver procedure or surgery, and we evaluated days from that procedure to the day HAP was found on the radiology report. HAP interventions and their complications were considered only for the index admission. We followed up the clinical status (alive or deceased) 1 year after the discovery of HAP. The presence of cancer, specified by type and stage, was also evaluated.

The literature search was done by searching PubMed/Medline database using the key words “hepatic artery pseudoaneurysm” to identify articles, including case reports and case series, previously published on this topic. By this strategy, we identified 55 cases from single case reports and case series as presented in Supplementary Table 1. All categorical variables were summarized with the number of patients and the percentage. Continuous variables were illustrated with a mean. Comparisons between patients alive and dead were completed using a Chi-square test for independence. Follow-up was at least 12 months, so a simple comparison was completed. We assume complete follow-up at 12 months. The numbers are too small for any adjustment for confounders. Analysis was completed with SAS version 9.

## Results

### Demographic data, etiology, and associated conditions

Out of 27 patients with diagnosis of HAP, 17 (63%) were male and 10 (37%) were female. The median age was 57 years (range: 25–87 years). Average BMI was 26.8 kg/m^2^. Baseline characteristics are mentioned in Table 1. Additional relevant cardiovascular risk profile, substance abuse and immunosuppressive/chemotherapy is summarized in Table 2. The etiology of HAP was postprocedural in 89.9% including: balloon angioplasty and TIPS (3 patients; 11.1%), liver transplantation (4 patients; 14.8%), liver biopsy (3 patients; 11.1%), laparoscopic cholecystectomy (1 patient, 3.7%), biliary procedures—ERCP and biliary drain placement (4 patients, 14.8%), Whipple procedure (3 patients; 11.1%), and trauma (4 patients; 14.8%). A minority of patients developed spontaneous HAP—10.1%. In patients where etiology of HAP was procedural, HAP developed on the average 27.7 days after the procedure. Malignancy was found in 52% of patients. Tumor invasion was present in 3 patients, of which one had undergone ERCP while the other 2 did not have any procedures. Hepatobiliary infections associated with HAP were seen in 14% of patients (4 patients) of which 2 had hepatobiliary abscess and other 2 had abscess and cholangitis. Etiology for all 27 cases is summarized in Table 3. Cirrhosis was seen in 7 patients; liver necrosis was found in 2 patients and 2 patients had associated Portal vein thrombosis (PVT) as illustrated in Table 1.

**Table 1:** Summary of demographics, comorbidities and laboratory results at the time of HAP diagnosis.

Demographic findings	
Age (mean)	56.4 years
	**Total number (percentage)**
Female /Male	10 (37.0%) /17 (63%)
**Facility**	
Arizona	3 (11.1%)
Florida	7 (25.9%)
Minnesota	17 (63.0%)
**Associated comorbidities /medications**	**Total number (percentage)**
Malignancy	14 (51.9%)
Anticoagulation Use	6 (23.1%)
Cirrhosis	7 (25.9%)
Liver Transplant	7 (25.9%)
Liver necrosis	2 (7.4%)
Portal vein thrombosis	2 (7.4%)
**Laboratory results**	**Mean**
Platelets (x10^9^/L)	217.1
White blood cell count(x10^9^/L)	13.7
Hemoglobin (g/dL)	10.28
Hematocrit (%)	30.85
AST (U/L)	138.2
ALT (U/L)	122.1
Total Bilirubin (mg/dL)	2.6
INR	1.4

**Table 2:** Summary of relevant cardiovascular risk profile, substance use and immunosuppression.

Patient characteristics	N (%)
Hypertension	14 (51.9%)
Hyperlipidemia	6 (22.2%)
Diabetes mellitus	4 (14.8%)
Smoking	
Current	6 (22.2%)
Former	7 (25.9%)
Never/Unknown	14 (51.9%)
Alcohol use	
Non-drinkers or unknown	13 (48.1%)
Occasional or former drinkers	7 (25.9%)
Regular drinkers	7 (25.9%)
Recreational drug use	
No drug use or unknown	24 (88.9%)
Former drug use	1 (3.7%)
Current drug use	2 (7.4%)
Immunosuppressive therapy/chemotherapy	
None	17 (63%)
Tacrolimus combination therapy (+Prednisone or Mycophenolate)	6 (22%)
Specific Regimens (CHOP, Gemcitabine/Abraxane, Denosumab)	3 (11%)
Prednisone alone	1 (4%)

**Table 3:** Summary of etiology of hepatic artery pseudoaneurysm.

Etiology of HAP	Count
Spontaneous	3
Post liver transplant	4
Post-surgery (non-liver transplant)	
Whipple’s procedure	2
Laparoscopic cholecystectomy	1
Post non-surgical procedural	
Biliary drain placement	3
Balloon angioplasty for hepatic artery stenosis	1
Liver biopsy	3
Transhepatic intrahepatic portosystemic shunt	1
Radiofrequency ablation of hepatic lesion	1
ERCP	1
Infection/tumor related	3
Trauma	4

### Location of HAP and clinical presentation

Of 27 patients, 23 patients (85.2%) had intrahepatic HAP (defined as HAP of left hepatic artery, right hepatic artery, middle hepatic artery), and 4 patients (14.8%) had extrahepatic HAP (HAP of common hepatic artery, proper hepatic artery and origin of hepatic artery). The right hepatic artery (RHA) was the predominant location for pseudoaneurysms in 19 patients (70.4%), the left hepatic artery (LHA) accounted for 3 cases (11.1%), while the remaining 5 cases (18.5%) were distributed among other locations: common hepatic artery (2 cases), middle hepatic artery (1 case), origin of the hepatic artery (1 case), and proper hepatic artery (1 case). 22% of patients (6 patients) had anatomical abnormalities of hepatic artery vascularization, including 5 with accessory left hepatic artery from gastric artery and 1 accessory left hepatic artery from superior mesenteric artery.

**Figure 1:**
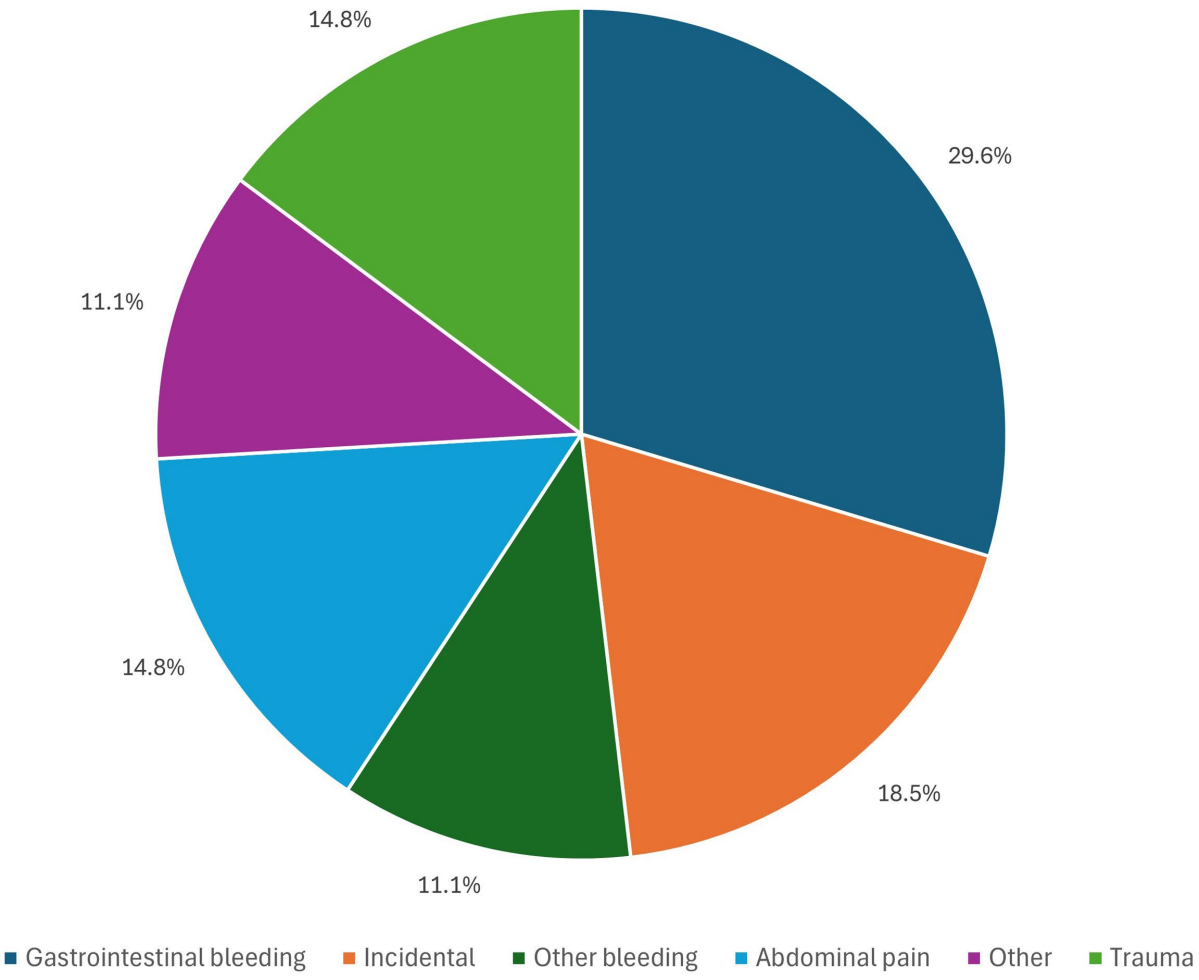
Presenting symptoms at the hepatic artery pseudoaneurysm diagnosis.

The most common presenting symptom was gastrointestinal (GI) bleeding, which was seen in 8 patients (29.6%), 3 patients (11.1%) had bleeding other than GI bleed (intraoperative brisk arterial bleeding through ileostomy during biliary stent removal by endoscopic retrograde cholangiopancreatography (ERCP); subcapsular hepatic hematoma). Abdominal pain was the presenting complaint for 4 patients (14.81%) while 5 patients (18.52%), where asymptomatic and HAP was discovered accidentally as illustrated in Figure 1. Presenting symptoms defined as “other” were reported by 4 patients (14.81%) and included: mycotic HAP (incidentally found during evaluation of infective endocarditis with hepatic and splenic abscesses); fever and weight loss; generalized weakness; fever.

### Complications and associated features

HAP rupture occurred in 17 patients (63.0%), mostly of RHA (64.7%). Of these 17 cases (58.8%) had GI bleeding. There were 2 reported hepatic arterioportal fistulas, 1 arteriovenous fistula, and 2 hepatic artery-bile duct fistulas. 14 patients (51.9%) required blood transfusion. Hemorrhagic shock was seen in 2 patients. Out of the six patients on anticoagulation, only 2 patients developed hemorrhagic shock. The first patient with hemorrhagic shock due to HAP rupture presented with massive hematemesis, melena and hypotension while on apixaban. This patient was successfully managed by IR coil embolization of proper hepatic artery secondary to malignancy related arterial injury. The second patient with hemorrhagic shock related to HAP rupture, had a history of Marfan’s syndrome and was on apixaban for atrial fibrillation, when he presented 2 weeks after a fall with symptomatic anemia and hemorrhagic shock due to large hepatic hematoma. This patient was treated with RHA pseudoaneurysm coil embolization and direct thrombin injection of RHA and LHA. Both patients that had hemorrhagic shock were on anticoagulation and had successful outcomes following HAP coil embolization. Hepatic necrosis was seen in 2 patients, portal vein thrombosis was seen in 2 patients, with 1 patient out of them having both PVT and liver necrosis. The overall infection rate was 3%.

### Orthotopic liver transplant recipients

Subgroup analysis of post-transplant HAP included 7 patients (26%) with a median age of 43 years of which 71% (5/7 patients) were men. HAP was incidentally found during post LT screening in 3/7 patients (42%). Only 1 patient died (14%; 1/7 patients), and he was the only one with concomitant infection. Since 2023, Mayo Clinic Florida has adopted a standard post LT protocol that includes assessing the transplant recipient’s hepatic arterial vasculature by Doppler ultrasounds within 24 h of the transplant and again on day 14. The previous protocol was to perform a Doppler ultrasound or CTA abdomen on post LT days 7 and 21 to confirm vascular patency.

### Operative management and outcome

Of 27 patients 2 patients did not have any specific IR intervention; 2 patients had stenting, 1 had glue embolization and 22 patients were treated with IR coil embolization. Of the 22 patients treated with IR coil embolization, 3 patients experienced treatment failure after the first attempt; however, treatment was successful after the second attempt. One patient had necrosis of the liver suspectedly due to HAP coil embolization and one patient developed post embolization syndrome. The 2 patients who did not undergo intervention, both had RHA pseudoaneurysm incidentally found on CT abdomen. The first patient’s HAP was diagnosed on surveillance CT abdomen after LT. The asymptomatic HAP was not treated by IR in favor of surveillance serial CTs of the abdomen which showed that HAP remained unchanged over the next 5 years. The second patient’s HAP was found incidentally on CT abdomen performed for hematuria following a renal biopsy. Since hematuria was self-limiting due to renal biopsy and HAP was incidentally found, no intervention for HAP was undertaken. This patient did not have surveillance imaging for monitoring of HAP and remained asymptomatic for the next 4 years.

Overall, 1 year survival was found to be 81.5%. There was no significant difference in survival in patients with or without GI bleed (80% vs. 82.4%, *p* = 0.879). Categories associated with increased mortality were: need for transfusion (*p* = 0.021), tumor invasion (*p* = 0.023), PVT (p = 0.02) and liver necrosis (p = 0.02). Categories not associated with increased mortality were: known HAP (*p* = 0.381), HAP rupture (*p* = 0.382), anticoagulation (*p* = 0.856), hemorrhagic shock (*p* = 0.25), liver cirrhosis (0.143), GI bleed (0.879), hepatobiliary abscess (*p* = 0.079), liver transplant (*p* = 0.738), spontaneous HAP (*p* = 0.381) and malignancy (*p* = 0.163) as seen in Table 4. Five patients (8.5%) died within 1 year of HAP diagnosis, however none of the deaths were attributed to HAP. Two of the five deceased patients experienced GI bleeding, however, these deaths were not directly attributed to GI bleeding in the context of HAP, rather, to progression of their underlying malignancy. Of the remaining patients who died, an additional two deaths were attributed to progression of advanced cancer, while one patient with traumatic HAP died due to pulmonary complications related to the traumatic event.

**Table 4:** Univariate analysis for survival at 1 year of HAP and associated conditions, medications and gender.

Variable	*p*-value
HAP associated with tumor invasion	0.023
Liver necrosis	0.002
Portal venous thrombosis	0.002
Transfusion	0.021
Gender (male)	0.879
Anticoagulated	0.856
Gastrointestinal bleed	0.879
HAP rupture	0.382
Hemorrhagic shock	0.25
Hepatobiliary abscess	0.079
Liver cirrhosis	0.143
Liver transplant	0.738
Known HAP	0.381
malignancy	0.163
Spontaneous HAP	0.381

## Discussion

HAP is a rare condition and is associated with high mortality after its rupture. Due to its rarity, publications are limited to case presentations, case series or reviews related to specific subgroups as illustrated in Supplementary Table 1. Our study is the largest retrospective study in the literature describing 27 patients with HAP from various etiologies, their clinical manifestations, management, and outcome.

HAP is commonly diagnosed after it ruptures and causes GI bleeding, while asymptomatic cases are usually discovered on routine post trauma screening or as a part of comprehensive post-transplant evaluation and monitoring. Although our study found that the need for transfusion in the setting of HAP led to increased mortality risk, hemorrhagic shock following HAP rupture occurred only in a small number of patients and resulted in favorable outcome following treatment with IR coil embolization. Most common location of HAP was found to be intrahepatic (85.2%), and only 14.8% were extrahepatic. These findings are different from what Marshall at co-authors described in 13 liver transplant recipients who found extrahepatic location to be more common (2). Our study points out that the most frequent location of HAP was on the RHA and that was the most frequent site of HAP rupture as well. Prior studies have not reported on this specific data. Due to the small sample size, we were unable to perform subgroup analysis regarding the outcome in relation to the HAP location.

Etiology of HAP is usually related to hepatobiliary and pancreatic procedures, infections, penetrating or blunt abdominal trauma, atherosclerosis, and an exceedingly small fraction are considered spontaneous (4–19). Possible underlying causes of spontaneous HAP could be atherosclerosis, remote history of abdominal trauma and lack of reporting those during history or during publication process, undiagnosed collagen disorders, variance in hepatic artery anatomy and arterial branching. HAP is a subclassification of splanchnic artery aneurisms (SSA), and its risk factors include diabetes mellitus, hypertension, hyperlipidemia, coronary insufficiency, smoking history, pregnancy and previous pancreatitis history (20). It is possible that these same risk factors play a role in the development of HAP especially in cases labeled as spontaneous. Due to the rarity of HAP and the ununified reporting and investigative process to determine the exact causes of spontaneous HAP in previous publications, future reporting should include the presence or absence of these risk factors and a detailed history. A retrospective study from Denmark, which included 259 non-operatively managed patients who sustained blunt abdominal trauma and had appropriate CT follow up, reported a 4% incidence of HAP following abdominal trauma which was not related to the degree and severity of liver injury (21). We describe comparable results with 89.9% of HAP being post-procedural, while the remaining 10.1% categorized as spontaneous. Spontaneous HAP is exceedingly rare but has been reported in several previous cases and tends to have an excellent prognosis (1, 3, 22). Similarly, all patients with spontaneous HAP in our institution had an excellent outcome.

HAP occurrence in patients with hepatobiliary and pancreatic malignancy is associated with biliary stent placement, liver biopsy, percutaneous biliary drainage, tumor invasion, or regionally applied chemotherapy which increase the risk of arterial wall damage, infection, and HAP development (4, 6–12, 15, 17–19). HAP can occur from 5 days to 2 years after biliary plastic stent placement as illustrated in the literature review on the topic of HAP rupture following biliary stent placement (16). Mucosal necrosis from cancer, intraluminal compression of the stent and effects of chemotherapy before and after stenting are thought to be the risk factors for HAP rupture in this setting. In our institution half of the patients with HAP had hepatobiliary or pancreatic malignancy, but that was not associated with increased mortality. Only in 5 cases (18%) HAP occurred after biliary stent placement.

HAP is a rare complication of liver transplantation (LT) procedures with an incidence of 0.3–2.6% (2, 13). Although a rare post-transplant complication, it comprised 26% of all HAP cases in our institution, which may be related to routine surveillance imaging required by this patient population leading to an increased rate of detection. HAP is usually diagnosed within 2 to 3 weeks, and up to 2 months after liver transplantation (2, 13). At Mayo Clinic HAP was diagnosed on an average 22 days (about 3 weeks) post LT. Incidental finding of HAP in post LT screening was higher in our institution, compared to previously reported data (43% vs. 23%) (2). Interestingly, compared to the reported high mortality rate of HAP in LT recipients 69–75% (2, 13), we observed significantly lower mortality of 15%. This observed difference might be related to the rates of infections, which were higher in previous studies compared to our findings. Concomitant infection in HAP in LT recipients was documented to be as high as 85% (2) with substantial number of fungal infections. Contrary to these findings, in our experience at Mayo Clinic, HAP associated with infection was seen in only one LT recipient (15%) which was also the only patient that died. These observed differences in outcome might be related to the expertise of the transplant center itself, improvement in IR techniques and improved surgical techniques and infection prevention over the years. Also, in our institution many incidental HAP diagnosed in routine post-transplant screening led to early and safe interventional radiology treatments which might have contributed to a significant decrease in mortality.

Treatment options for HAP are embolization and stent graft placement. Various embolic agents can be considered depending on anatomic considerations, coagulation profile, and operator expertise. When abscess is present caution should be exercised with stent graft placement to prevent long-term graft infection. Super-selective embolization with microcatheter technique is recommended to prevent non-target embolization. These are typically complex patients, and close follow-up is recommended to monitor for complications and HAP recurrence or incomplete treatment. Ruptured HAP has higher chances of post treatment complications. Out of 25 interventions, we had 3 initial treatment failures that required reintervention, and 2 patients had suspected procedural complications. Despite a few unsuccessful first attempts at HAP treatment, all of them had successful IR reintervention.

The mortality rate after HAP rupture may be as high as 70% (3). HAP with intraperitoneal hemorrhage has been shown to have the highest mortality rate of 82% (3). Most of the previously published data were case presentations or case series and the two largest studies of HAP were related to LT recipients. Previously published data describing HAP related to various etiologies including LT, trauma, biliary stent placement, pancreaticoduodenectomy, percutaneous biliary drainage, splenectomy, gastrectomy, hemi-pancreatectomy, liver resection, idiopathic, reported an overall mortality rate of 34.5% (Supplementary Table 1) (23–30). The overall mortality rate of HAP in Mayo Clinic was 18.5%, despite almost 65% of cases having ruptures. In our study we did not find that HAP rupture, therapeutic anticoagulation, gastrointestinal bleeding, and hemorrhagic shock were associated with increased mortality. In contrast, risk factors associated with increased mortality in our study were need for transfusion, HAP associated with tumor invasion, presence of PVT and liver necrosis. Of the 5 patients (8.5%) who died within 1 year of HAP diagnosis none of the deaths were attributed to HAP. Four of the five deaths were attributed to progression of the underlying malignancy, and while two of these four cases experienced GI bleeding, this was not directly related to HAP. The remaining patient with traumatic HAP died due to pulmonary complications related to the traumatic event. Overall infection rate in our cohort was low, 3%, which is the most prominent difference from previously reported data on patients with HAP and might have had an impact on a higher one-year survival of our cohort compared to previous studies.

### Study limitations

While this is the largest retrospective study on this topic, it is limited by a small sample size (27 cases) due to the low incidence of HAP, which reduces statistical power, increases the likelihood of type II errors, and prevents the use of stratified subgroup analyses. Although data were collected from three different Mayo Clinic sites including Florida, Minnesota, and Arizona, this retrospective analysis within a single hospital network may still be susceptible to selection bias. Additionally, as Mayo Clinic functions as a referral center, complete patient information may not always be available at time of admission for patients transferred from outside facilities. Relying solely on documented records may lead to incomplete clinical profiles, potentially impacting the comprehensiveness and accuracy of the data used in this study. Retrospective studies can pose certain risks of selection and reporting bias, and this study is no exception. Lastly, there is a risk of publication bias by past authors as previous literature has predominantly reported severe cases.

## Data Availability

The original contributions presented in the study are included in the article/Supplementary material, further inquiries can be directed to the corresponding author.
