# Supplementary material for: Hepatic artery pseudoaneurysm–the Mayo Clinic experience and literature review
Source: Front Med (Lausanne). 2024 Dec 10;11:1484966. doi: 10.3389/fmed.2024.1484966 (PMC11666383; doi:10.3389/fmed.2024.1484966)
Supplement: Supplementary file 1 [file Data_Sheet_1.docx]

Supplement 1. List of previously published literaturre on hepatic artery pseudoaneurysm

| case number | author | HAP etiology | timeframe of HAP diagnosis | spontaneous | alive | age | infection | location | presenting symptoms | cancer |
| --- | --- | --- | --- | --- | --- | --- | --- | --- | --- | --- |
| 1 | Briceno (2) | resenction of cholangiocarcinoma | 2 years after procedure | no | yes | 78 | no | RHA | GI bleed | cholangiocarcinoma |
| 2 | Ostreballe (1) | traumatic liver injury | 5 days | no | yes | 25 | / | / | incidental | no |
| 3 | Ostreballe (1) | traumatic liver injury | 5 days | no | yes | 20 | / | / | incidental | no |
| 4 | Ostreballe (1) | traumatic liver injury | 5 days | no | yes | 45 | / | / | incidental | no |
| 5 | Ostreballe (1) | traumatic liver injury | 5 days | no | yes | 9 | / | / | incidental | no |
| 6 | Ostreballe (1) | traumatic liver injury | 5 days | no | yes | 30 | / | / | hemodinamic failure, acute anemia | no |
| 7 | Ostreballe (1) | traumatic liver injury | 5 days | no | yes | 42 | / | / | hemodinamic failure, acute anemia | no |
| 8 | Ostreballe (1) | traumatic liver injury | 5 days | no | yes | 42 | / | / | incidental | no |
| 9 | Lloret Estan (3) | splenectomy after trauma | 6 months | no | yes | 18 | no | SMA or UMA | abdominal pain and GI bleed | no |
| 10 | Samaras-case 1 (4) | right lobe hemihepatectomy with HA chemiotherapy and wedge resection 1 year later | 15 days | no | yes | 62 | / | hepatic artery | abdominal pain | rectal carcinoma(liver metashasis) |
| 11 | Samaras-case 2 (4) | right lobe hemihepatectomy with HA chemiotherapy and wedge resection 1 year later | 1 year | no | yes | 38 | yes | LHA | GI bleed | multi-focal hepatocellular carcinoma |
| 12 | Kharel (8) | gastrectomy | 10 days | no | yes | 58 | yes | common hepatic artery | GI bleed, abd pain and fever | gastric cancer |
| 13 | Inoue (9) | biliary stent placement (plastic) | 20 days | no | yes | 68 | yes | hepatic artery | GI bleed and fever | Klatskin’s tumor |
| 14 | Nezu (10)-case 1 | biliary stent placement (plastic) | 5 days | no | yes | / | / | / | / | / |
| 15 | Nezu (10)-case 3 | biliary stent placement (plastic) | 1 day | no | yes | / | / | / | / | / |
| 16 | Monroe (11) | biliary stent placement (metalic) | 3 weeks | no | yes | / | / | / | / | / |
| 17 | Wutanabe (12) | self expandible metalic stent (SEMS) | 9 months | no | yes | 70 | yes | RHA | Abdominal pain | adenocarcinoma |
| 18 | Hyun (13) - case 1 | SEMS for malignant biliary obstruction | 152 days | no | yes | 61 | / | GDA | melena | pancreas head tumor |
| 19 | Hyun (13) - case 2 | SEMS for malignant biliary obstruction | 15 days | no | no | 65 | / | GDA | melena | metastasis from colon cancer |
| 20 | Hyun (13) - case 3 | SEMS for malignant biliary obstruction | 76 days | no | no | 51 | / | GDA | melena | pancreas head tumor |
| 21 | Hyun (13) - case 4 | SEMS for malignant biliary obstruction | 40 days | no | loss to follow up | 79 | / | GDA | melena | CBD cancer |
| 22 | Hyun (13) - case 5 | SEMS for malignant biliary obstruction | 15 days | no | no | 65 | / | GDA | hematochezia | GB cancer |
| 23 | Hyun (13) - case 6 | SEMS for malignant biliary obstruction | 152 days | no | no | 72 | / | RHA | hematemesis | Klatskin's tumor |
| 24 | Marshal (14)-case 1 | liver transplant | 26 days | no | yes | 43 | No | / | Incidental | / |
| 25 | Marshal (14)-case 2 | liver transplant | 60 days | no | yes | 43 | No | / | Pain, acute anemia | / |
| 26 | Marshal (14)-case 3 | liver transplant | 132 days | no | no | 43 | yes | / | Incidental | / |
| 27 | Marshal (14)-case 4 | liver transplant | 31 days | no | no | 43 | yes | / | Incidental | / |
| 28 | Marshal (14)-case 5 | liver transplant | 15 days | no | no | 43 | yes | / | Rupture | / |
| 29 | Marshal (14)-case 6 | liver transplant | 27 days | no | no | 43 | yes | / | Rupture | / |
| 30 | Marshal (14)-case 7 | liver transplant | 22 days | no | yes | 43 | yes | / | Intra abdominal bleed | / |
| 31 | Marshal (14)-case 8 | liver transplant | 52 days | no | no | 43 | yes | / | Fever, acute anemia | / |
| 32 | Marshal (14)-case 9 | liver transplant | 8 days | no | yes | 43 | yes | / | Intra abdominal bleed | / |
| 33 | Marshal (14)-case 10 | liver transplant | 16 days | no | no | 43 | yes | / | GI bleed | / |
| 34 | Marshal (14)-case 11 | liver transplant | 68 days | no | no | 43 | yes | / | GI bleed | / |
| 35 | Marshal (14)-case 12 | liver transplant | 75 days | no | no | 43 | yes | / | Rupture | / |
| 36 | Marshal (14)-case 13 | liver transplant | 30 days | no | no | 43 | yes | / | Intra abdominal bleed | / |
| 37 | Michel (21)-case 1 | liver transplant | / | no | no | / | yes | / | / | / |
| 38 | Michel (21)-case 2 | liver transplant | / | no | no | / | yes | / | / | / |
| 39 | Michel(21)-case 3 | liver transplant | / | no | no | / | yes | / | / | / |
| 40 | Michel(21)-case 4 | liver transplant | / | no | no | / | yes | / | / | / |
| 41 | Kim (15) | splenectomy and splenic artery embolization | 6 years ago | no | yes | 56 | / | aberant hepatic artery off the superior mesenteric artery | Melena and hematemesis | chronic lymphocytic leukemia |
| 42 | Martinez Ramos (16) | interventional radiology percutaneous biliary drainage | 13 days | no | yes | 70 | / | RHA | Abdominal pain and GI bleed | cholangiocarcinoma |
| 43 | Reiter (17) | none | N/A | yes | yes | 41 | / | RHA | Abdominal pain | / |
| 44 | Sandelis-Pérez (18) | None | N/A | yes | yes | 77 | / | LHA | Abdominal pain | / |
| 45 | Satoh (19)-case 1 | SEMS | 14 days | no | no | 72 | no evidence | RHA | fever, jaundice, anemia | gastric cancer |
| 46 | Satoh (19)-case 2 | SEMS | 7 days | no | no | 70 | not suggested | pancreatoduodenal artery | fever, Abdominal pain, melena | cholangiocarcinoma |
| 47 | Sreh (20) | None | N/A | yes | yes | 84 | Yes | RHA | Abdominal pain and GI bleed | / |
| 48 | Vernadakis (22) | pancreaticoduodenectomy | 6 days | no | yes | 55 | / | RHA | GI bleed | Pancreatic head cancer |
| 49 | Rai R (23) | Biliary stenting | 2 years | no | yes | 47 | yes(cholangitis) | RHA | Melena | lymphoma |
| 50 | Park JY(24) | Biliary stenting | 1 month | no | yes | 62 | yes(cholangitis) | LHA | fever, jaundice | hilar cholangiocarcinoma |
| 57 | Inchingolo R (28) | Biliary stenting | 1 month | no | yes | 75 | / | GDA | melena, abdominal pain | pancreatic cancer |
| 58 | Chun JM- (29) | Biliary stenting | / | no | yes | 47 | / | RHA | melena | no |
| 59 | Yasuda M (30) | Biliary stenting | 1 year | no | yes | 78 | / | RHA | hematemesis | no |
| 60 | Ding Z (31) | Biliary stenting | 13 days | no | yes | 56 | / | LHA | hematemesis | no |
| 61 | Yamauchi K (32) | Biliary stenting | 14 days | no | yes | 78 | / | LHA | jaundice, fever | no |
